# “As soon as you’ve had the baby that’s it…” a qualitative study of 24 postnatal women on their experience of maternal obesity care pathways

**DOI:** 10.1186/s12889-016-3289-1

**Published:** 2016-07-22

**Authors:** Sarah Dinsdale, Kay Branch, Lindsay Cook, Janet Shucksmith

**Affiliations:** Health and Social Care Institute, School of Health and Social Care, Teesside University, Middlesbrough, TS1 3BA UK; Women and Children Centre, The James Cook University Hospital, South Tees Hospitals NHS Foundation Trust, Marton Road, Middlesbrough, TS4 3BW UK; Public Health, Middlesbrough Council, PO Box 502, Vancouver House, Gurney Street, Middlesbrough, TS1 9FW UK

**Keywords:** Pregnancy, Postnatal, Obesity, Care pathway, Teachable moment

## Abstract

**Background:**

Maternal obesity is associated with risks to mother and infant, and has implications for healthcare costs. United Kingdom (UK) levels of maternal obesity are rising, with higher prevalence in North East (NE) England, where this study was set. Pregnancy is often seen as an opportune time for intervention – a ‘teachable moment’ - which is ripe for promoting behaviour change. In response to rising obesity levels, a National Health Service (NHS) Foundation Trust in NE England implemented three maternal obesity care pathways contingent on Body Mass Index (BMI) at time of booking: pathway 1 for those with BMI ≥30 kg/m^2^; pathway 2 for BMI ≥35 kg/m^2^; and pathway 3 for BMI ≥40 kg/m^2^. These incorporated relevant antenatal, intrapartum and postnatal clinical requirements, and included a focus on weight management intervention. This evaluation explored the accounts of postnatal women who had been through one of these pathways in pregnancy.

**Methods:**

The study used a generic qualitative approach. Semi-structured interviews were carried out to explore the views and experiences of 24 recent mothers (aged 20–42), living in NE England, who had commenced on one of the pathways during pregnancy. Interviews explored experiences of weight management support during and after pregnancy, and perceived gaps in this support. Data were analysed using thematic content analysis.

**Results:**

Three main themes emerged reflecting women’s views and experiences of the pathways: communication about the pathways; treating obese pregnant women with sensitivity and respect; and appropriate and accessible lifestyle services and information for women during and after pregnancy. An overarching theme: differences in care, support and advice, was evident when comparing the experiences of women on pathways 1 or 2 with those on pathway 3.

**Conclusions:**

This study indicated that women were not averse to risk management and weight management intervention during and after pregnancy. However, in order to improve reach and effectiveness, such interventions need to be well communicated and offer constructive, individualised advice and support. The postnatal phase may also offer an opportune moment for intervention, suggesting that the simple notion of seeing pregnancy alone as a window of opportunity or a ‘teachable moment’ should be reconsidered.

**Electronic supplementary material:**

The online version of this article (doi:10.1186/s12889-016-3289-1) contains supplementary material, which is available to authorized users.

## Background

The United Kingdom (UK), in common with other developed countries, is experiencing increasing rates of maternal obesity, defined as a Body Mass Index (BMI) >30 kg/m2 among women at the start of pregnancy [1–3]. Predictors of maternal obesity include socio-economic deprivation, age, parity and ethnicity [1, 4, 5]. Data from the North East (NE) of England reflect this rising trend, and indicate that the region has higher rates than the UK average [4, 5]. Maternal obesity is associated with various health risks to both mother and infant, including diabetes, hypertensive disorders, pre-eclampsia, pre-term delivery, late foetal loss and stillbirth [6–9] as well as having implications for healthcare resources [10]. The literature highlights that there is also the potential for post-pregnancy weight retention, continuing into subsequent pregnancies [11, 12].

Pregnancy is increasingly perceived as a ‘teachable moment’ in the public health field, with the suggestion being that it is an appropriate time for weight management intervention, both because women are going through a life transition (and may be prepared to re-evaluate their habits and priorities), coupled with having frequent contact with healthcare professionals [12]. In line with the above, in the UK two sets of national guidance exist for weight management in pregnancy [13, 14] and these provide recommendations for healthcare professionals around advice, support and information for obese pregnant women. Furthermore, obesity is recognised in the Clinical Negligence Scheme for Trusts (CNST) 2013/14 Maternity Clinical Risk Management standards as a risk that Trusts must manage consistently against minimum standards [15].

Evidence around the effectiveness of weight management interventions in pregnancy is, however, unclear. Some have reported a lack of statistically significant effects on weight gain in pregnancy, or on pregnancy outcomes for the mother or baby [16, 17]. However others report benefits including reductions in weight gain in pregnancy, a reduced prevalence of associated co-morbidities, and improved mental health [18–22].

An emerging body of literature is considering the viewpoints and experiences of obese pregnant women themselves in relation to their healthcare and weight management during pregnancy [16, 23–30]. Such studies provide key learning for the design and implementation of interventions. For example qualitative syntheses [16, 26] have reported women receiving limited, inconsistent or confusing advice in relation to weight management leading to concerns around the risks of changing diet or activity levels in pregnancy, and indicating a need for interventions to consider women as individuals, recognising their own social context, pre-pregnancy behaviours and attitudes. Within NE England, Heslehurst et al. [27] considered women’s experiences in relation to an antenatal weight management programme. Qualitative interviews highlighted the complexities of women’s relationships with their weight, and their priorities in pregnancy, including the baby’s health, appropriate nutrition, and limited weight gain. These priorities could be used to motivate women to engage with weight management services antenatally. Through exploring the experiences of pregnant women and recent mothers, other research has also highlighted the fact that the postnatal phase may be an opportunity for women to make changes to their lifestyle, whilst noting that more understanding into this period is required [29, 30].

Within a National Health Service (NHS) Foundation Trust in NE England, three care pathways were developed for the management of maternal obesity. These pathways, based on published national guidelines and research evidence, and the expertise of a multi-disciplinary steering group, were as follows: pathway 1 for women with a booking BMI ≥30; pathway 2 for women with a booking BMI ≥35; and pathway 3 for women with a booking BMI ≥40. These pathways were set up with the aim of providing the appropriate level of antenatal intervention to manage risks associated with obesity in pregnancy, as well as reducing known barriers to healthcare professionals’ management of maternal obesity [31]. The pathways were provided alongside a written maternal obesity guideline, and acted as a tick list for healthcare professionals incorporating antenatal, intrapartum, and postnatal clinical requirements relevant to the booking BMI, as well as an opportunity for weight management intervention. A full copy of the pathways for each BMI category is provided (see Additional file 1). Women on pathways 1 and 2 received relevant checks (e.g. an oral glucose tolerance test) and lifestyle support and advice (e.g. information on healthy diet and physical activity) via routine antenatal care. For women with a BMI ≥40 there a specific service, the ‘healthy lifestyles clinic’ was provided, which involved a consultation with a Midwife Consultant, followed by a dietitian appointment; women were also offered 2 extra scans. Women were invited to attend up to four healthy lifestyle clinics throughout pregnancy. Postnatally, women on all three pathways should be signposted to general weight management or lifestyle services in their local community.

The early implementation of these pathways has been evaluated via a mixed methods study [31], in which. Pregnant women highlighted positivity about the pathways when they were not seen as a tick box process, although women with a BMI <40 that weight management support and advice was lacking. This earlier study also highlighted that pregnant women and healthcare professionals felt that postnatal support was important; however audit data indicated this was currently lacking.

This current study evaluated the pathways at a later point in time, with a different sample of women. Importantly it involved women who had been through the pathways in their entirety and were then in a postnatal phase. This adds further insight into what support women received both in pregnancy and postnatally, the importance women placed on this support, and what they felt would be of value. Furthermore a solely qualitative study of this nature allowed for the exploration of in-depth views and accounts of the women recruited to this study, regarding their experiences of the pathways. As pathways 1 and 2 differed from pathway 3 in terms of the level of support provided these in-depth accounts also allowed for consideration of women’s experiences in relation to their own weight status and subsequent BMI pathways.

## Methods

This study aimed to explore and gain understanding of recent mother’s general views and experiences of the maternal obesity care pathways. Due to known differences between the pathways the study also aimed to consider differences in opinion between women on pathways 1 or 2 and pathway 3 where evident.

The overall research question was broad and exploratory: ‘what were women’s views and experiences of the maternal obesity pathways’?

Specific research questions were:

- What did women think of the services and support provided antenatally as part of the pathways? What worked well or not so well? Were there any gaps in this support?
- What did women think of the services and support provided in the postnatal period as part of the pathways? What worked well or not so well? Were there any gaps in this support?
- What did women perceive to be the barriers and facilitators to accessing services and support provided antentally/postnatally?
- Did women implement any lifestyle changes during pregnancy and/or after pregnancy as a result of the pathways?
- Were there any differences in the views of women on pathways 1 or 2 and 3?

### Research team

The research team came from a range of academic and clinical disciplinary backgrounds including; psychology, sociology, public health, nutrition, and midwifery.

### Design

This was a commissioned evaluation and therefore used a generic qualitative design, also referred to as ‘qualitative description’. This inductive approach, is used for more pragmatic research, such as answering policy and practice questions. It is not aligned to, or guided by an established approach or set of philosophical assumptions, instead aiming to understand events and the meanings individuals give to them and convey these to the reader [32–34].

### Sampling and recruitment of participants

A stratified random sampling strategy was used. The Midwife Consultant at the Trust acted as gatekeeper, having access to a database detailing all women who had a booking BMI of ≥ 30 kg/m2 and who had commenced on one of the three pathways. Women who had given birth 3–9 months prior to this evaluation’s sampling strategy being developed (Jan 2013) were initially invited to take part. A sample size of approximately 30 women was aimed for; therefore invitations were sent to 120 women to account for potential low recruitment rates. The research team randomly selected 120 cases from an anonymised version of this database: 60 cases from pathways 1 and 2 (because of similarities in these two pathways); and 60 cases from pathway 3. Women who had experienced adverse pregnancy outcomes (miscarriage, late fetal loss or stillbirth), women under 16 years of age at the time of booking, and women who could not speak or read English were excluded from recruitment.

Postal information and pre-paid reply slips were sent out on behalf of the research team in April 2013. On reply slips women were asked to provide their postcode, age, whether this was their first baby, and their contact details. Postal information was the same for pathways 1, 2, and 3; however reply slips were printed on different colours to enable identification of the pathway followed on response. In total 13 women responded from the initial 120 invitations and 11 went on to take part (9 % recruitment rate). Reminders were sent out to non-responders (*n* = 107) in May 2013. 7 further women responded and took part (raising the recruitment rate to 15 % for the first round).

A second round of postal recruitment was commenced in June 2013. A further 60 women with a with a BMI ≥30/35 from the original dataset were randomly selected. Alongside this, 51 women with a BMI ≥40 were selected from a dataset covering a more recent delivery period (given birth 3–7 months prior to the second round of sampling and recruitment) due to limited numbers in the original data set. In total 11 women responded to the second round of recruitment and 6 went on to take part (5.4 % recruitment rate). The women who showed interest but did not participate in the study either stated a lack of time to participate, or were uncontactable despite several attempts.

In total 24 women took part in the evaluation (11 with a BMI ≥30/35, and 13 with a BMI ≥40). Participant ages ranged from 20–42, with the majority aged between 30–34. For seven women this was their first baby; the remainder (*n* = 17) had more than one child. Postcode information provided by the participants enabled the research team to assign individuals to a corresponding Index of Multiple Deprivation (IMD) national deprivation quintile [35]. The majority of participants (*n* = 18) resided in areas with a national deprivation quintile of 1 or 2 (with quintile 1 being the most deprived). See Table 1.

**Table 1:** Participant characteristics

Participant	BMI group	Pathway	Age	Parity	National deprivation quintile calculated using Indices of Multiple Deprivation (IMD) 2010 data [35] (Quintile 1 [Q1] = most deprived)
Participant 1	≥30/35	1/2	30	Multiparous	Q2
Participant 2	≥30/35	1/2	34	Primiparous	Q2
Participant 3	≥30/35	1/2	29	Multiparous	Q2
Participant 4	≥30/35	1/2	30	Primiparous	Q2
Participant 5	≥30/35	1/2	28	Primiparous	Q2
Participant 6	≥30/35	1/2	39	Multiparous	Q1
Participant 7	≥30/35	1/2	34	Multiparous	Q5
Participant 8	≥30/35	1/2	36	Multiparous	Q1
Participant 9	≥30/35	1/2	32	Primiparous	Q1
Participant 10	≥30/35	1/2	38	Multiparous	Q5
Participant 11	≥30/35	1/2	32	Multiparous	Q1
Participant 12	≥40	3	20	Primiparous	Q1
Participant 13	≥40	3	24	Primiparous	Q1
Participant 14	≥40	3	34	Primiparous	Q2
Participant 15	≥40	3	27	Multiparous	Q2
Participant 16	≥40	3	28	Multiparous	Q1
Participant 17	≥40	3	35	Multiparous	Q2
Participant 18	≥40	3	29	Multiparous	Q3
Participant 19	≥40	3	42	Multiparous	Q3
Participant 20	≥40	3	34	Multiparous	Q1
Participant 21	≥40	3	35	Multiparous	Q2
Participant 22	≥40	3	34	Grande-Multiparous	Q1
Participant 23	≥40	3	33	Multiparous	Q4
Participant 24	≥40	3	33	Multiparous	Q4

### Data collection

Semi structured one to one interviews were carried out between April and August 2013, in the women’s’ chosen environment (women’s homes *n* = 20; via telephone *n* = 4). Written consent was obtained from all participants before being interviewed. The majority of women interviewed at home had their baby/children present (*n* = 15), six also had a partner/friend/ family member(s) present.

All data collection was carried out by the same female researcher (SD), who was educated to Master of Science (MSc) level, and experienced in qualitative research, including in the field of maternal obesity. Prior to each interview the researcher made contact with participants to introduce herself and the research and answer any questions. Participants were informed that the researcher worked for a University, and was not from a clinical background or linked to the maternity services they had accessed. They were informed that the interviews were part of a commissioned evaluation to understand the experiences of women in relation to the care and support they had received in relation to their weight, during and after pregnancy.

Interview schedules were designed to explore women’s views and experiences in relation to the pathways. Topics included: BMI/ weight measurements, obesity communication, being on the care pathways and lifestyle support and advice received during pregnancy, engagement with any services offered, and the postnatal period. Interview topics, developed in consultation with the wider team, were broad to enable women’s discussion of personal salient issues. The first two interviews served as a pilot, and further themes for exploration were developed iteratively as they became apparent. Recruitment to the study ceased once data saturation was agreed between the research team. Interviews ranged in length between 26 and 91 min.

### Data analysis

All interviews were digitally recorded with the consent of participants, anonymised and fully transcribed. Field notes were also made for each of the interviews. In two cases notes were taken as the participant did not wish to be recorded. Interview data were analysed using Thematic Content Analysis [36], using QSR NVIVO software. One researcher (SD) led on this phase, involving open coding of all transcripts and supporting field notes, and the development of themes and subthemes. Analysis was on-going throughout data collection, allowing consideration of new interview themes and recognition of data saturation, which was agreed when no new findings were coming out of the research. Regular team discussions enabled discrepancies, emerging themes, data saturation and final themes to be discussed and agreed, acting as a verification strategy and ultimately strengthening the rigour of findings.

Three themes were identified to describe women’s views and experiences of the pathways. These themes aimed to provide a rich description of the data, identifying key issues for women due to the broad exploratory nature of the overall research question [37], and encompass findings in relation to the specific research questions presented in the methods section. These were: ‘Communication about the pathways’ , ‘Treating obese pregnant women with sensitivity and respect’ , and ‘Appropriate and accessible lifestyle services, and information for women’. The subthemes that these overarching themes encompass are detailed in Table 2. Throughout all themes a clear difference in the level of care, support and advice given to women on pathway 3 in comparison to pathways 1 and 2 was apparent, and is presented as an overarching theme.

**Table 2:** Themes and subthemes

Overarching theme: ‘Differences in care, support and advice across pathways’	
Themes	Sub-themes
‘Communication about the pathways’	• Awareness and understanding • Clinical aspects of the pathway (including risk communication)
‘Treating obese pregnant women with sensitivity and respect’	• Women’s views on the approach used by Healthcare Professionals • Recognition of the individual
‘Appropriate and accessible lifestyle services, and information for women’	• Engagement with the Healthy Lifestyle Clinic • Weight management aspects of the pathways and subsequent lifestyle changes • Postnatal diet and lifestyle advice received • Sustained lifestyle changes after pregnancy

## Results

The following themes were generated to describe women’s views and experiences in relation to the pathways, in particular addressing what worked well or not so well with regards to the services and support (received both antenatally and postnatally), barriers and facilitators to accessing support, perceived gaps in support and changes implemented as a result of the pathways. It became clear throughout all data that there was a clear difference in the level of support and advice given to women on pathway 3 in comparison to pathways 1 and 2, and this is echoed within each of the themes.

### Communication about the pathways

Clear differences were evident between the perceptions of women on pathway 3, and those on pathways 1 and 2 in terms of the level of communication about the pathway and its implications for their pregnancy. Their understanding and awareness of the fact that they were receiving some kind of intervention also differed. Women on pathway 3 described being very clearly aware that they were on the pathway, through seeing a copy of this in their hand-held notes or recalling discussions around this throughout their pregnancy. Most were aware that the ‘clinical checks’ they received throughout pregnancy were often because of their BMI, and for the safety of themselves and the baby as they were at a ‘higher risk’.

*“They explained that I was on the pathway for extra monitoring, because of a raised BMI, just obviously some of the risks to do with larger babies or whatever, extra monitoring to check for diabetes and things.* (Participant 24, Pathway 3)

Conversely, almost all women on pathways 1 and 2 described being unaware that they were on a pathway at all, and those that did know often demonstrated confusion about what this meant. Most of these women felt that the pathways had not been sufficiently explained, and when the topic of pathways was raised this was brief, and only at the beginning of pregnancy. Most of these women wanted a better explanation to keep them informed, to act as an incentive to being healthy, or because they felt this would be good practice. For some, being offered clinical checks was the only way of identifying that they were on a pathway, as this may have differed from the service they had received in previous pregnancies. However, some did not associate clinical checks with their BMI and questioned if they were routine for everyone.

“*Let people know that they are on a pathway, and just give like a bit more detail about the pathway, what it means for the rest of your pregnancy. I mean I know midwives are restricted, with the time they’ve got as well…but I think that would be more useful.”* (Participant 1, Pathway 1/2)

Risk communication features on the pathways; and women on pathway 3 often recalled risks being carefully explained. Those on pathways 1 or 2 generally were not aware of specific risks due to their BMI, due to a lack of explanation, or discussion of risks only occurring when a complication arose. Women perceived how other issues (e.g. breastfeeding) were given priority. In the absence of an explanation women described finding out about risks from friend’s experiences or via the internet. Women perceived being told about risks positively, preferring healthcare professionals to be honest and upfront, and also wanting constructive advice about how to minimise these risks.

### Treating obese pregnant women with sensitivity and respect

Some women recalled concern about being judged in relation to their weight, due to previous personal experiences or friends’ experiences. However, women on pathway 3 were very positive about the approach used by healthcare professionals to discuss the issue.

*“My friend, she’s slightly overweight and when she went (to her antenatal appointments) they were saying to her you need to do this, you need to do that, you’re too big…So I was quite nervous for a couple of weeks leading up…but as soon as I (got there) she (midwife) was lovely, they were very positive, there was never a bad word said about it… I felt like it wasn’t just me, a lot of people were the same, it wasn’t my fault and they were giving me ways to solve it, which was good.”* (Participant 12, Pathway 3)

There were a few instances where women with a BMI over 40 recalled being made to feel ‘naughty’ , ‘embarrassed’ or ‘ashamed’ because of their weight, due to healthcare professionals being ‘blunt’ or insensitive, or women perceiving that weight was discussed too much.

*“Just stop going on about it quite as much as they do. Say it once or twice, but constantly being ****severely obese **** and BMI, BMI, BMI - it’s kind of drummed into your head all the time.”* (Participant 15, Pathway 3)

There were some women on pathways 1 or 2 who felt their weight had been ‘skimmed over’ , or written in notes but never discussed, due to the midwife’s fear of offending, or limited midwife time. Some women discussed how talking about weight was important.

*“On one of my scans it said ‘difficult viewing because of obesity’ …but no one ever mentioned it. I read it and I was like, oh God! But I was pleased that no one actually said it. But I suppose they shouldn’t skirt around it… (they should) be more supportive, and bring it up. I think they are too polite almost; they daren’t say it.”* (Participant 11, Pathway 1/2)

Women discussed the words used by healthcare professionals when talking about weight. BMI was the term most often used, and most preferred as it was ‘less frightening’ and ‘non-judgemental’. Most said they understood what this meant through engagement with commercial slimming clubs, or the media. However some did express a lack of personal understanding, or felt that others would not understand it.

*“(BMI is) nice and diplomatic I think. For me it’s a measurement; it’s a medical thing, nobody is saying you’re fat…I was happy that they used that because I thought that that keeps everyone the same … I understand what it means…but there might be people out there who don’t really understand what that means. As long as that’s explained to them I suppose…”* (Participant 2, Pathway 1/2)

Some women described how the terms ‘obese’ , ‘clinically obese’ or ‘morbidly obese’ had been used during their pregnancies, and perceived these words negatively, especially when used among first time mothers at their booking appointment. Some described how weight was a sensitive issue anyway and they were likely to be feeling more vulnerable and emotional during pregnancy.

*“I said to my husband ‘I’m obese’, and he went, ‘no you’re not’. I went, ‘well, that’s what I’m classed as’… I think she could have said, ‘Your BMI is high; we’ll just keep an eye on it.’ I just think that the word itself is an awful word to use…I think they could find a different word, majorly overweight or something like that, just so it sounds a bit friendlier…I think it makes people feel fat and horrible.“*(Participant 5, Pathway 1/2)

Often women recalled a personal history of struggling with their weight. When discussing their own weight women often used self-deprecating humour, put themselves or their level of control down, and adopted terms such as ‘huge’ , ‘big’ , ‘fat’ , ‘massive’ , ‘wobbling’ , ‘fatty’ , or ‘podgy’ , or ‘I used to be beautiful’. Some women traced increasing weight gain back to previous pregnancies, or certain difficult periods in their life. Some described how, within society, or among healthcare professionals there was perceived stigma and assumptions in relation to obesity, and expressed concern about being judged because of their weight or being on the pathways. Among these women, some described how they were essentially an individual, and that varying degrees of obesity, varying knowledge levels, and their complex histories around weight should consistently be acknowledged by healthcare professionals, rather than simply using their obesity as an ‘essentialising’ stigma [38].

*“I’ve come home and I’ve thought, that’s not fair, you’re assuming just because I’m overweight. My overweightness is because of a bad patch in my life. I’d put on a massive amount of weight and I found it really hard to get it back off. It’s not that it continues. But people assume that because you’re big that it’s because of what you’re eating now. It’s not necessarily because I’m putting weight on, I’m staying the same. I’m just not losing it…Trying to get to the bottom of that would be helpful, finding out why people eat as much as they eat, or why they eat the bad things they eat, because you never get support to help yourself with that sort of side, like the mental psychological side.”* (Participant 23, Pathway 3)

### Appropriate and accessible lifestyle services, and information for women

The majority of women on pathway 3 had attended all or some of the healthy lifestyle clinic appointments (*n* = 9). Lack of engagement or partial engagement was due to women being exempt from the service due to comorbidities, not being offered the service, being unable to attend the appointments due to a timing issue, missing appointments due to an early birth, an unknown pregnancy, or delays in the referral. Women who had attended the healthy lifestyle clinic often perceived this service positively, and found the extra level of care reassuring. The few negative perceptions related to practical issues such as the timing of appointments, or the availability of dietetics support.

Most women on pathway 3 who had attended the healthy lifestyles clinic viewed advice on diet and activity as useful, especially when it was tailored to them and their personal challenges. Some changes to lifestyle during pregnancy were evident among these women through making ‘healthy swaps’ , eating regular meals, and limited weight gain. Some of these women wanted additional tips and recipes for healthy eating alongside their family. Among these women the activity advice received had largely focused around walking or aqua-natal exercise. Most wanted more information about available activity services, or for there to be specific services for expectant mothers.

*“When I spoke to the dietitian she gave me like a plan of ways to substitute things that I would normally eat and have something else instead…like brand stuff that you can swap for less calories in, which was good to know.”* (Participant 12, Pathway 3)

Among women who had not attended the clinic, or were on pathways 1 or 2, dietary advice was often not provided, focusing solely around foods to avoid in pregnancy. When advice was provided it was perceived as ‘limited’ or ‘generic’ , or, at times, conflicting, with women recalling being told ‘don’t eat for two’ , ‘eat your five a day’ or being given written resources to replace a verbal explanation. Similarly physical activity information was minimal, and only provided when women sought this themselves. Minimal lifestyle changes were recalled among this group. Fears about the safety of the baby were viewed as a major barrier to participating in physical activity in pregnancy, particularly among primparous women. Some described giving regular exercise up upon becoming pregnant, and ‘wrapping themselves in cotton wool’. Some women questioned whether midwives had time to provide information on diet and activity, felt they assumed women understood a healthy lifestyle, or prioritised other issues (e.g. breast feeding).

*“I did try water aerobics once, but I had to look for it myself, and I didn’t know how much pressure you can put on. Obviously that was my first [baby]… I didn’t know what was safe to be doing…so in the end I think I didn’t bother.”* (Participant 10, Pathway 1/2)

These women described wanting information about safe activity in pregnancy, diet plans for them and their family, portion sizes, the amount of extra calories needed, or support to deal with personal food challenges, (e.g. binge eating). They felt that women’s understanding of a healthy lifestyle should be assessed and information tailored as a consequence. Women felt information could come from the midwife, a dietitian, or a service specifically designed for pregnant mums that would allow people to address diet and activity in a peer group setting, to share experiences and advice together.

*“I think even if they did a sort of class for early pregnancy, so you knew what was coming, what you’ve got to look forward to, how you can help yourself… You’re all together, often asking the same questions anyway.”* (Participant 4, Pathway 1/2)

When asked about making changes, women across the pathways described how during pregnancy their bodies would dictate to them, to some extent, the diet and lifestyle they could follow. Some perceived that they could eat what they wanted during pregnancy, ‘enjoy being pregnant’ and focus on weight loss afterwards. Conversely some women explained that during pregnancy they were more ‘conscious’ about eating healthier as they were nurturing a baby. Others experienced cravings for certain foods, were constantly hungry, needed to ‘graze’ , experienced sickness, felt full during pregnancy, or experienced extreme tiredness or discomfort, preventing engaging in more activity.

*“I just embraced being pregnant…I just loved it and I thought what goes on can come off again… if you want two pieces of cake, then you will have it.”* (Participant 9, Pathway 1/2)

The pathways also stipulate that women should be provided with healthy lifestyle advice and signposted to community weight management postnatally. The majority of women recalled little in the way of information or advice about weight management for the postnatal period. A minority felt that the information received in pregnancy would be of use to them now, or were provided with information on postnatal services and support (however these were not perceived as particularly accessible).

Women talked at length about desired postnatal advice and support recalling the difficulties of ‘having a different body’ , experiencing weight gain and difficulties with weight loss (especially among multiparous women), struggles with being healthy whilst caring for a demanding new baby, a desire to embed a healthy lifestyle within their family, a desire to lose weight before a subsequent pregnancy, and a perception that advice provided often focused solely around the baby. Others described how risks were continuously emphasised during pregnancy but felt that support around these issues ceased when the baby was born.

*“So you’re on this BMI pathway, this important thing that they have to discuss with you, and then as soon as you’ve had the baby that’s it, it’s just like forgotten about, but if it was important then, it should be important now I think.*” (Participant 24, Pathway 3)

In the absence of information and support there were multiple accounts of women seeking information on services and support themselves, and making efforts, with varying success, to change their behaviour. Women perceived the timing of postnatal advice as a key consideration; during pregnancy or immediately after birth women said they felt vulnerable, emotional and that the baby’s needs took priority. Information should therefore be provided once they returned home and had settled into some kind of routine, on discharge from the community midwife or up to 3 months postnatally. Women described how advice could come from a health visitor, the dietitian, a specific service aimed at helping new mums, or at their GP surgery.

Women provided information on the types of services and support that they would find most useful, such as advice on ‘healthy meals for busy mums’ , or details of community exercise classes available. Group sessions were frequently discussed positively, providing an opportunity to socialise and share tips, and do exercise in a comfortable environment. These opportunities could be tagged onto other services at children’s centres, enabling women to involve their children or to exercise whilst they had childcare facilities.

*“If this was a class to lose baby weight, especially for mums that have just had a baby…then you’re not going to be stood next to some lanky person in Lycra who looks gorgeous… But if it was all mams wobbling together, I’d feel better about it.”* (Participant 3, Pathway 1/2)

Women often spoke of having busy lives, with children to prioritise, and wanted services that were local and easily accessible around existing commitments. Costs of participating in classes were viewed as a major barrier, and free taster sessions or vouchers to incentivise engagement were suggested.

## Discussion

This research followed on from an earlier evaluation of the same service [31], and adds support to a number of previous findings, with a different group of women, at a time point where the pathways had been implemented for a longer duration. However, importantly it adds a number of additional insights to this work. Perhaps most significantly this research involved women who had given birth, and therefore experienced the pathways in their entirety, also providing insight into women’s experiences postnatally. Furthermore, a focus on eliciting purely qualitative accounts, with an inductive approach to analysis (as opposed to a mixed methods paper, where a deductive approach was used for analysis of qualitative data) allowed for more in-depth insight and detail regarding women’s experiences of the pathways, and in relation to their own weight. In particular this study repeatedly emphasised the differences in experiences in relation to pathways 1, 2 and 3 which provided an overarching theme across the results and influenced the take home messages described below.

Through the exploration of women’s views and experiences of the maternal obesity pathways both during and after pregnancy it was clear that in general women in this sample were not averse to risks being discussed or weight management intervention. However, this study demonstrated the importance of effective communication and constructive advice and support.

Four key take home messages were evident and have been discussed in turn here.

1. There is a need for effective communication of the pathways in all the monitored BMI groups
2. There is a need to ensure that obese women are treated with sensitivity and respect, acknowledging that women’s lives and reasons for weight gain are complex
3. There is a need for appropriate and accessible information and linkage with accessible services for women (antenatally and postnatally), and possible service redesign in pathways 1 and 2
4. There is a need to recognise that pregnancy can be a teaching and learning opportunity, but pregnancy may not be the best moment for women to be able to make changes.

Women on pathways 1 and 2 were often unaware that they were receiving any type of intervention. Women’s desire for improved communication supports earlier research advocating the importance of women feeling they are not merely part of a tick box exercise [31]; poor communication about care can lead to feelings of anxiety and unpreparedness for complications [24]; and women want to be informed because they see the baby’s welfare as a priority [27], and are aware of risk discourses surrounding pregnancy [39, 40].

Findings also demonstrated the importance of a sensitive and individualised approach. Stigma in relation to obesity in pregnancy is recognised within the literature [23, 24, 41]. Lupton [39] explained how ‘problematic behaviours’ attract moral judgement, regardless of any compelling reason for these behaviours. Women in this study wanted their own knowledge and behaviours to be recognised, and disliked being categorised as ‘obese’ , supporting research that women were keen to be ‘seen as a person behind the fat’ [23]. Anticipated stigma, or a ‘one size fits all’ approach could potentially prevent engagement among some women, supporting the importance of personalised information reported elsewhere [20]. Additionally the importance of terminology was evident, with the word ‘obese’ commonly being seen as offensive, especially due to the negative connotations linked to obesity more generally [23].

Perceived midwife discomfort in talking about weight may have been a result of an expectation that midwives would raise the issue as part of routine practice without relevant support. Others have reported midwives’ concerns that raising the topic of obesity might alienate women from further engagement with midwifery services, emphasising a need for midwife training to improve confidence in sensitively raising and discussing weight [42].

Among women on pathways 1 and 2 lifestyle advice was described as lacking, and they were therefore too concerned about safety to make changes or maintain previous physical activity habits, reflecting findings reported elsewhere [16, 26, 28, 43]. It became somewhat evident that the intervention for women on pathways 1 and 2 was an ‘add-on’ to midwives’ current busy workload, raising questions about its value and purpose. In order to support these women, services would need to be remodelled, undoubtedly bringing cost implications. However, there are cost implications in not delivering an effective intervention. Recognising a lack of NHS resources available to support pregnant women with weight management, recent research has considered the feasibility of group sessions for pregnant women co-facilitated by midwives and a commercial weight management organisation [44]. A wider trial based on this approach is currently underway [45].

The exploration of the viewpoints of postnatal women in the UK in relation to postnatal support and advice received is a relatively new area of consideration, to which this study adds. In a meta-synthesis of obese women’s maternity experiences, Smith and Lavender [30] concluded that the postnatal phase appeared to be a time when women are ready to make lifestyle changes, but noted that wider exploration was needed to understand why, and what would help women. This current research demonstrated a strong sense of a gap in postnatal information, an overwhelming desire for more support, and increased motivation for postpartum weight loss, a finding reported elsewhere [29, 30, 46].

Despite women’s weight often being an enduring issue, potentially being carried into future pregnancies, women described feeling that support simply stopped when they had given birth. This provides direct research support for the claim that pregnancy is too often treated as a medicalised issue, set in a context of ‘risk’, with most priority focused on the fetus and producing a healthy baby rather than on the mother [39, 40, 47]. Furthermore findings support recent research with pregnant women who perceived a fragmentation between ‘me’ and ‘my pregnancy’ , a distinction influenced by the biomedical discourses surrounding pregnancy with the fetus being perceived as a separate entity [28].

The consideration of the viewpoints of postnatal women is important in designing a postnatal intervention that meets their needs. Women are reported to face numerous barriers to adopting a healthy lifestyle after pregnancy, despite motivation [48–50], and therefore interventions should be consider timing, availability, accessibility, and women’s support needs. This current study additionally highlighted the desire of post-natal women for peer group sessions both during and after pregnancy so that they felt comfortable, and were among similar others.

Evidence of women making changes to their behaviour during pregnancy due to the advice they received was limited to a few women on pathway 3. Interestingly others described changes due to simply being pregnant, or their baby being their priority, supporting other research that women give the health and safety of their unborn baby priority [27], or carry out behaviours due to a perceived responsibility to provide the ideal gestational environment for the fetus [28]. Such findings support the thesis that, for some women at least, pregnancy acts as a ‘teachable moment’ [12] and is therefore an appropriate time to raise weight and provide intervention. This is often for pragmatic reasons - these are generally healthy women in frequent contact with service and all the many opportunities that offers for contact, persuasion and surveillance. However, there is another thread to this characterisation of the ‘teachable moment’ which relates to the assumption that women – at this special time in their lives, on the threshold of producing a new human being – a fresh start - will be uniquely motivated to change behaviour. Lupton [39, 40] argues that societally women are expected to engage in monitoring and regulation of their own behaviours to reduce risk and protect this unborn child, and as a result some women do become vigilant in making changes in order to conform to what society portrays as a ‘good mother’ who does not ‘contaminate’ her baby in anyway.

However, importantly some women described how physiological changes or cravings in pregnancy dictated their behaviours or prevented behaviour changes. Campbell et al. [16] describe how pregnancy is viewed as a ‘transient and translational time’ , with dietary cravings, nausea and discomfort shaping behaviour. Padmanabhan et al. [28] highlight that pregnancy can be a time of conflict between carrying out healthy behaviours for the benefit of the baby, whilst also seeing pregnancy as a time to relax previously adhered to rigid rules, or experiencing challenges in adopting healthy behaviours due to cravings, tiredness, concerns about the risks of activity, or busy lifestyles. Furthermore, a recent meta-synthesis [51] highlights that for some women pregnancy is perceived as a period where they have a lack of control over their body, and they may strive to regain this control postnatally. Indeed some women within this research described how interventions in the postnatal phase would be more valuable as they had other priorities and concerns during pregnancy. Other research has suggested this, when exploring the views of obese pregnant women or obese women trying to conceive [25] This supports the findings of other research [29, 30] showing that postnatal women are keen to make changes to their lifestyle.

Returning to the notion of pregnancy as ‘a teachable moment’ [12] this raises the important issue of whether this concept should be more critically considered. Olander et al. [50], for instance, helpfully describe how considering the perinatal period through a ‘Capability-Opportunity-Motivation Behaviour’(COM-B) framework, rather than treating it as a blanket opportunity, suggests that there are various potential opportune intervention periods, and that the potential for behaviour change fluctuates throughout this pregnancy and postpartum period. The COM-B framework, developed via synthesis of 19 existing frameworks of behaviour change, proposes that behaviour change depends on these three necessary determinants [50, 52].

This current paper supports a critical stance in relation to the concept of a teachable moment, indicating that the postpartum period might be an equally or more effective ‘teachable moment’ for some women, and posing the question as to whether the ‘teachable moment’ should be viewed as being longer in duration. This argument has been alluded to in a recent article aimed at guiding clinical practice [53]. Rather than seeing pregnancy as a short window of opportunity, intervention between pregnancies would recognise the potential for reducing post-pregnancy weight retention [11, 12]. Women in this study described being motivated to change due to troublesome experiences during this pregnancy, and in order to have a more positive experience in future pregnancies. In this sense these individuals may be a more captive audience for successful intervention. Such an approach would also reflect a recommended shift towards more comprehensive, women-centred interventions [47], by acknowledging women’s own need for support, rather than merely losing track of them after the baby is born.

Recommendations from this evaluation supported the need for improved antenatal lifestyle advice for women on the lower pathways, more direct information about postnatal support, and the need for training for midwives to support implementation of the pathways. Training for community midwives has since been provided, alongside revised pathways for ease of use. Additional tailored advice surrounding diet and weight gain is also being provided to women. Postnatally further links have been established with existing exercise services.

### Limitations

Since women were no longer engaging with the maternity services postal recruitment was chosen as the most appropriate way to contact them; however recruitment was slow. This may have been influenced by the opt-in nature of recruitment, alongside the sensitivities surrounding obesity, and women being busy with a new baby to care for.

A self-selecting sample could have resulted in biased recruitment of women who had extreme perspectives on their care. However, among participants this was very rarely the case. All but one woman interviewed were of white ethnicity, and it would be of value to hear the viewpoints of other ethnic groups. However this sample reflects the ethnic make-up of this part of NE England [54]. Recruitment achieved an almost equal number of participants from the lower and higher pathways, and representation across different demographic factors; age, parity, and Socio Economic Status (SES). Findings from this sample of women were therefore perceived to be generalisable due to the qualitative nature of the study, whereby generalisability refers to the extent to which theory developed within one study may be exported to provide explanatory theory for experiences of other individuals in comparable situations [55]. In 6 interviews a family member, friend or partner was also present, due to the participant opting for this to be the case. Although this could influence the interview dynamic, in the majority of cases this actually facilitated participant comfort, and did not appear to hinder discussion.

## Conclusions

Findings from this research indicated that overweight and obese women are not averse to risk management and weight management interventions during pregnancy; however their feedback provides important insight into how weight management interventions during and after pregnancy can be more successful. Comparing the experiences of women across the different pathways indicates that interventions should be well communicated, based on a sensitive and individualised approach, and need to incorporate appropriate and accessible information and support both during and after pregnancy. Importantly, among some women the postnatal phase may be an equal or in some cases better opportunity for intervention, suggesting that the concept of pregnancy as a unique window of opportunity as a ‘teachable moment’ should be carefully considered. Tailored support for women during pregnancy and postnatally would help to convince women that their own needs were being considered as well as those of the baby.

## Abbreviations

BMI, Body Mass Index; CNST, Clinical Negligence Scheme for Trusts; COM-B, Capability-Opportunity-Motivation Behaviour; IMD, Indices of Multiple Deprivation; MSc, Master of Science; NE, North East; NHS, National Health Service; Q, Quintile; SES, Socio Economic Status; UK, United Kingdom

## Additional file

Additional file 1: Maternal obesity care pathways according to BMI (2012). (PDF 252 kb)
