# Supplementary material for: “As soon as you’ve had the baby that’s it…” a qualitative study of 24 postnatal women on their experience of maternal obesity care pathways
Source: BMC Public Health. 2016 Jul 22;16:625. doi: 10.1186/s12889-016-3289-1 (PMC4957370; doi:10.1186/s12889-016-3289-1)
Supplement: Additional file 1: — Maternal obesity care pathways according to BMI (2012). (PDF 252 kb) [file 12889_2016_3289_MOESM1_ESM.pdf]

Additional file 1: Maternal obesity care pathways according to BMI (2012)

Visit	Action<br>Antenatal	√ Tick or add when complete or N/A		
		BMI<br>30-34.9	BMI<br>35-39.9	BMI<br>≥40
**Booking**	Calculate and record BMI<br>**TICK** appropriate BMI pathway to be followed √ — Wt<br>BMI:<br>→			
	Explain Body Mass Index (BMI)			
	Book for maternity team based care<br>Refer to:<br>-High Dependency Antenatal Clinic if other co-morbidities present			
	-Healthy Life style clinic if BMI ≥40 & **no** co-morbidities present (Site X only)			
	Booking BMI≥35 advised to deliver in obstetric led unit			
	Discuss Healthy Life Style information leaflet/web pages			
	Initiate discussion on weight management in pregnancy			
	Exercise advise given			
	Offer Folic acid 5mg			
	Offer & discuss Vitamin D 10[illegible]g<br>(Healthy Start Women's Vitamins)			
	**Book OGTT screening on :**<br>**Previous gestational diabetes**<br>Screen 1. at 16-18weeks<br>Screen 2. at 28 weeks<br>**All other women**<br>Screen between 24-28weeks			
	**SITE Y- BMI 30-35 for RBS at booking not for OGTT**			
	Complete VTE risk assessment			
	Complete Thyroid function test			
	Discuss the risks of raised BMI -document possible intrapartum complications			
	Use appropriate BP cuff document size			
**High dependency care / ANC**	Discuss weight management and exercise in pregnancy			
	Undertake MRSA screening as per protocol.			
	Review VTE score and need for enoxaparin			
	Complete alert card in hand held notes: BMI, antenatal plan, delivery plan, all pathways			
	Arrange 20 week anomaly scan			

Visit	Action<br>Antenatal	√ Tick or add when complete or N/A		
		BMI<br>30-34.9	BMI<br>35-39.9	BMI<br>≥40
**20 wks**	USS for fetal wellbeing			
	Provide infant feeding information			
	Review all pathways			
	Discuss signs & symptoms of pre-eclampsia & advice line			
	Refer for Anaesthetic consultation			
	Arrange USS for 32 & 36 weeks			
	Check GTT is booked			
	RBS result (SITE Y)			
	Follow up investigation results			
	Discuss weight management and exercise in pregnancy			

Additional file 1: Maternal obesity care pathways according to BMI (2012)

**32-34 wks**	USS for growth and fetal wellbeing			
	Weigh and calculate weight gain<br>Antenatal examination			
	Individual manual handling risk assessment and tissue viability issues – advice and action plan			
	Discuss weight management and exercise in pregnancy			
	Follow up investigation results			
	Review all pathways			
**36-38 wks**	USS for growth and fetal wellbeing			
	Weigh and calculate weight gain<br>Antenatal examination			
	Discuss weight management and exercise in pregnancy			
	Follow up investigation results			
	Review all pathways			
	Review and discuss Anaesthetic labour/delivery management plan			
	Discuss possible intrapartum complications			
	Ensure infant feeding check list has been fully completed			
	Positions in labour discussed to support normal birth/ birth plan			
	Advise to withhold enoxaparin at onset of labour or day of LSCS			
	Liaise with labour/theatre unit if booked for IOL or LSCS to ensure appropriate equipment available			

**Action**<br>**Labour**	**√ Tick or add when complete or N/A**		
	**BMI 30 -34.9**	**BMI 35-39.9**	**BMI ≥40**
Place of delivery –Low dependency- follow low dependency protocols			
Place of delivery – High dependency			
If high dependency with no other risk factors can follow low dependency protocols on delivery suite			
Insert IV cannulae if Antenatal Thromboprophylaxis administered, H/O APH or previous PPH obtain FBC, group & save			
BMI> 40 insert IV cannulae<br>FBC, group & save			
IV cannulae commence VIP chart			
Continuous CTG monitoring, may need FSE			
Inform duty anaesthetist need for anaesthetic review			
Inform consultant obstetrician of admission			
Ranitidine as per protocol			
Pressure care guidelines			
TED stocking to be worn throughout labour			
Active management of 3rd stage of labour recommended and documented in notes			
If LSCS delivery -commence LSCS pathway			

**Action**<br>**Postnatal**	**√ Tick or add when complete or N/A**		
	**BMI 30-34.9**	**BMI 35-39.9**	**BMI ≥40**
Individualised postnatal plan of care documented			
Record observations as per guidelines			

Discuss analgesia requirements			
Postpartum thromboprophylaxis assessment, encourage early and regular ambulation			
If LSCS delivery -complete caesarean section checklist for inspection of wounds proforma- to support strict attention to wound care			
Provide bladder care as per guideline			
Continue Vitamin D 10[illegible]g<br>(Healthy Start Women’s Vitamins) if Breastfeeding			
Contraceptive advice offered			
Provide pre conceptual information on commencing folic acid 5 mgs 1 month before stopping conception			
Healthy life style advice given<br>Sign post to community weight management services			
Opportunity to talk about birth offered			
Discharge check list completed			
